# Lecithin and cardiovascular health: a comprehensive review

**DOI:** 10.1186/s43044-024-00523-0

**Published:** 2024-07-13

**Authors:** Moyinoluwa Comfort Onaolapo, Olubunmi Dupe Alabi, Oyedayo Phillips Akano, Bolade Sylvester Olateju, Lateef Olabisi Okeleji, Wale Johnson Adeyemi, Ayodeji Folorunsho Ajayi

**Affiliations:** 1 Department of Physiology, Ladoke Akintola University of Technology, PMB 4000, Ogbomoso, Oyo State Nigeria; 2 Anchor Biomed Research Institute, Ogbomoso, Oyo State Nigeria; 3 Department of Nutrition and Dietetics, Ladoke Akintola University of Technology, Ogbomoso, Oyo State Nigeria; 4 Department of Physiology, Babcock University, Ilishan Remo, Ogun State Nigeria; 5 Department of Nutrition and Integrative Physiology, Florida State University, Tallahassee, USA; 6 Obafemi Awolowo University Teaching Hospital, Ile-Ife, Osun State Nigeria; 7 Department of Physiology, Adeleke University, Ede, Osun State Nigeria

**Keywords:** Lecithin, Lipids, Cardiovascular health, Atherosclerosis, Lecithin cholesterol acyltransferase (LCAT), Reverse cholesterol transport (RCT), Low-density lipoprotein (LDL), High-density lipoprotein (HDL), Antioxidant

## Abstract

**Background:**

Cardiovascular diseases are one of the prime causes of mortality globally. Therefore, concerted efforts are made to prevent or manage disruptions from normal functioning of the cardiovascular system. Disruption in lipid metabolism is a major contributor to cardiovascular dysfunction. This review examines how lecithin impacts lipid metabolism and cardiovascular health. It emphasizes lecithin's ability to reduce excess low-density lipoproteins (LDL) while specifically promoting the synthesis of high-density lipoprotein (HDL) particles, thus contributing to clearer understanding of its role in cardiovascular well-being. Emphasizing the importance of lecithin cholesterol acyltransferase (LCAT) in the reverse cholesterol transport (RCT) process, the article delves into its contribution in removing surplus cholesterol from cells. This review aims to clarify existing literature on lipid metabolism, providing insights for targeted strategies in the prevention and management of atherosclerotic cardiovascular disease (ASCVD). This review summarizes the potential of lecithin in cardiovascular health and the role of LCAT in cholesterol metabolism modulation, based on articles from 2000 to 2023 sourced from databases like MEDLINE, PubMed and the Scientific Electronic Library Online.

**Main body:**

While studies suggest a positive correlation between increased LCAT activities, reduced LDL particle size and elevated serum levels of triglyceride-rich lipoprotein (TRL) markers in individuals at risk of ASCVD, the review acknowledges existing controversies. The precise nature of LCAT's potential adverse effects remains uncertain, with varying reports in the literature. Notably, gastrointestinal symptoms such as diarrhea and nausea have been sporadically documented.

**Conclusions:**

The review calls for a comprehensive investigation into the complexities of LCAT's impact on cardiovascular health, recognizing the need for a nuanced understanding of its potential drawbacks. Despite indications of potential benefits, conflicting findings warrant further research to clarify LCAT's role in atherosclerosis.

## Background

Lecithin, a vital component derived from both plant and animal sources [1], is a natural amalgamation of diglycerides comprising palmitic, stearic, with oleic acids joined with the choline ester of phosphoric acid [2]. It is a compound found naturally in various tissues of animals and plants, including egg yolks, soybeans and peanuts. Simple oil from soybeans contains 2 to 3 percent lecithin, and significant quantities can also be found in whey and corn oil [3]. It is a complex mixture of phospholipids, triglycerides and glycolipids. Phospholipids are the primary component of lecithin and are responsible for its unique properties. Notably, it is ubiquitous on the outermost layer of plasma membranes [4, 5] and has been identified as a substantial phospholipid in amniotic fluid and lung surfactant [6, 7].

Lecithin offers potential health benefits, including cognitive enhancement through its choline content [8–10], liver protection from toxins [9, 11, 12] and potential cardiovascular improvements by lowering cholesterol levels [13–16]. It also has widespread applications in the food industry, where it acts as an emulsifier, stabilizer, wetting agent and antioxidant, commonly found in various products [3, 17]. Additionally, lecithin plays a significant role in the industrial sector; it is utilized in paints, cosmetics, pharmaceuticals and textiles for its properties as an emulsifier, wetting agent, dispersant and lubricant [18–20].

Cardiovascular diseases (CVD) encompass a variety of disarrangements acting on the blood vessels and the heart. Prominent types of CVD consist of atherosclerosis, coronary artery disease, arrhythmias and heart failure [21]. These conditions contribute to the global burden of CVD-related morbidity and mortality. CVD risk factors are multifaceted and encompass lifestyle elements such as unhealthy diet, uncontrolled intake of alcohol, stress, smoking and insufficient physical activity [22]. Genetic predisposition, age and pre-existing medical conditions like hypertension and diabetes also influence CVD susceptibility. Fact from WHO states that CVD is the dominant root of death globally with approximately 17.9 million mortalities adjudged to CVD in 2016 [23]. Understanding these diverse CVD types and their risk factors is important for growing worthwhile prevention and treatment strategies.

Atherosclerosis stands as one of the most life-threatening cardiovascular diseases, affecting individuals as early as 20 to 29 years old [24]. It is characterized by the accumulation of plaques in large and medium arteries, primarily composed of cholesterol, fibrin and calcium [25]. Key lipid-related cardiovascular threats include increased levels of low-density lipoprotein (LDL), elevated plasma triglycerides and reduced high-density lipoprotein (HDL) [26]. Remarkably, lecithin plays a pivotal role by diminishing excess LDL, the "bad cholesterol," and promoting the synthesis of HDL, the "beneficial cholesterol."

Literature reveals lecithin cholesterol acyltransferase's (LCAT) pivotal role in anti-atherogenic reverse cholesterol transport (RCT) [27]. LCAT translates unbound cholesterol in emerging HDL molecules to cholesteryl esters, enhancing HDL's cholesterol transport capacity [28]. LCAT elevates HDL cholesterol and triglyceride-rich lipoproteins levels and reduces LDL particle size [29]. These conflicting roles call for further investigations into LCAT's influence on HDL metabolism, LDL size and their implications for optimizing cardiovascular health [28].

Despite the promising role of LCAT activity in decreasing LDL particle size, further investigations are warranted to clarify the precise mechanisms by which increased LCAT activity results in a decrease in LDL particle size. This will advance our understanding of the association between lecithin and atherosclerosis.

This review unravels the effects of lecithin in cardiovascular health and highlights the role of LCAT in modulating cholesterol metabolism. Given the persistent global threat posed by cardiovascular diseases, particularly atherosclerosis, comprehending the mechanisms connecting lecithin, LCAT, and cardiovascular health is crucial. This review provides insights for future research and clinical interventions aimed at mitigating the risk factors associated with atherosclerosis and related cardiovascular conditions.

## Search strategy

The scheme of this narrative review is to recount the potential of lecithin in cardiovascular health and highlight the role of LCAT in modulating cholesterol metabolism. This review was executed by searching for articles in MEDLINE, PubMed and the Scientific Electronic Library Online databases using the Medical Subject terms “lecithin,” “lecithin” and “cardiovascular health,” “LCAT” and “modulation of cholesterol metabolism.” Articles published during the period 2000–2023 in English were put together.

For article selection, clear criteria were established to ensure that only relevant studies were included in the review. Articles were chosen if they directly addressed the relationship between lecithin and cardiovascular health or discussed LCAT's role in modulating cholesterol metabolism. The focus was on articles published between 2000 and 2023 to capture recent findings, and consideration was given exclusively to studies published in English due to language limitations. Both experimental studies and clinical trials were eligible for inclusion to provide a comprehensive overview of the topic. Articles that did not meet these criteria or were duplicates, conference abstracts, reviews without original data or focused on unrelated topics were excluded. Screening of articles was conducted based on titles, abstracts and full texts, when necessary, with eligibility determined by two independent reviewers. Any disagreements in article selection were resolved through discussion or consultation with a third reviewer to reach a consensus. This systematic approach ensured that only relevant articles were included, enhancing the transparency and reproducibility of the review.

### Lecithin

Lecithin, identified as egg yolk lecithin, soybean lecithin, soybean phospholipid [2], lecithol, phosphatidylcholine [30], choline phosphoglyceride [31] and phospholutein [32, 33], has a phosphatide or phospholipid component which is found in naturally occurring substances from both plant and animal sources [1]. It occurs naturally as a blend of diglycerides composed of stearic, oleic and palmitic acids, associated with the choline ester of phosphoric acid [2]. In nature, lecithin can have phosphoric acid linked to glycerol in either the alpha or beta position [34].

**Fig. 1:**
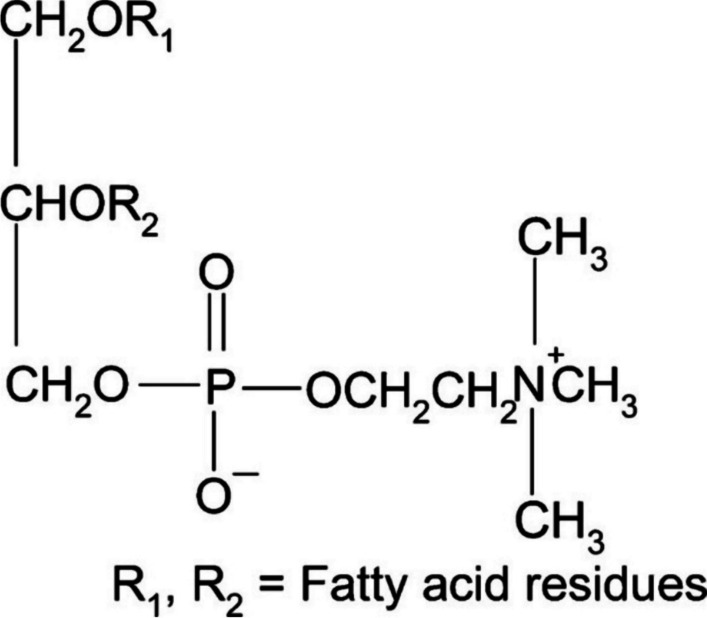
Chemical structure of lecithin

As a phosphatide present in virtually all living organisms, lecithin constitutes a substantial component of both brain and nervous tissue. It accounts for more than 50 percent of the phospholipids in most cell membranes of mammals [35]. Lecithin is also found on the surface of plasma membranes, forming the outermost layer [36]. The chemical structure of lecithin is displayed in Fig. 1.

Reports have indicated that lecithin is a significant phospholipid in amniotic fluid and lung surfactant [37–44]. Lecithin constitutes about 2–3% of the total weight of crude soybean oil and has also been found in substantial amounts in corn and wheat oils. Notably, lecithin makes up about 10% of the components of egg yolk [44–46].

Lecithin's major property of regulating cholesterol levels is a pivotal aspect of its role in promoting cardiovascular health [47–49]. It achieves this regulation through its capacity to reduce excess low-density lipoprotein (LDL), often dubbed as "bad cholesterol." High levels of LDL are associated with an increased risk of atherosclerosis and heart disease [50, 51].

Simultaneously, lecithin facilitates the synthesis of high-density lipoprotein (HDL), recognized as the "beneficial cholesterol" [52]. HDL contributes substantially to the removal of excess cholesterol from the blood circulation, transporting it to the liver for excretion, thus contributing to a healthier cardiovascular profile [53]. An increased presence of HDL is linked to a reduced risk of cardiovascular diseases [53].

Studies, such as the one conducted by Brunet and associates in 2003, have shown that diets rich in lecithin stimulate the secretion of bile acids by enhancing the formation of mixed micelles, which facilitate the solubilization and excretion of cholesterol. This mechanism involves elevated levels of phospholipids and cholesterol compared to diets lacking lecithin [47]. This, in turn, underscores lecithin's significance in maintaining a balanced cholesterol profile and supporting heart health.

Lecithin, a complex mixture of phospholipids, is subject to various chemical reactions, and its breakdown through hydrolysis is a fundamental one [54]. When subjected to complete hydrolysis, lecithin molecules disintegrate into their basic building blocks. This process yields two fatty acid molecules, often including palmitic, oleic and stearic acids [54], a molecule of phosphoric acid, glycerol, and a basic nitrogenous compound, choline [53]. These constituents are vital in understanding the composition and functional properties of lecithin.

Another intriguing chemical behavior of lecithin is its propensity to spontaneously bind with oxygen when exposed to atmospheric air [55]. This phenomenon is attributed to the double bonds present in the unsaturated fatty acids which are found within the triglyceride and phosphoglyceride components of lecithin. These double bonds are vulnerable to oxidation, which can lead to the formation of oxidative products such as epoxides and hydroperoxides [55]. This oxidative reactivity of lecithin has implications for its use in various applications, especially in the food industry and pharmaceuticals, where maintaining product stability and quality is of paramount importance [55]. Lecithin's oxidative reactivity finds various applications in the food sector, functioning as an emulsifier in baked goods and confectionery, stabilizing salad dressings and sauces, and aiding in the mixing of fats and oils in margarine. In therapeutics, it enhances drug solubility, bioavailability and stability in lipid-based drug delivery systems and supplements, while also regulating the release of medications in sustained-release formulations [55]

### Global incidence of cardiovascular disease

Cardiovascular diseases (CVDs) present a formidable global health challenge, annually claiming millions of lives across the globe [56]. This overarching term comprises of variety of conditions, such as coronary heart disease, stroke and peripheral artery disease, where the intricate interplay of cholesterol and its counterparts often stands at the heart of the matter [57].

Cholesterol, indispensable for cell membranes and various biological functions [58], becomes problematic when low-density lipoprotein (LDL) cholesterol rises to excessive levels, significantly contributing to the development of cardiovascular diseases (CVDs) [59]. Its vital role in cellular structure ensures membrane integrity and fluidity, facilitating essential cell functions and communication. Moreover, as a precursor for steroid hormones, bile acids and vitamin D synthesis, cholesterol plays a crucial role in hormonal regulation, digestion and overall metabolic processes [58. However, dysregulated LDL cholesterol levels can lead to the accumulation of plaque in arteries, contributing to the pathogenesis of CVDs [59]. The accumulation of LDL cholesterol in arteries results in the formation of plaques, which narrow the lumen of blood vessels and impede the flow of blood, thereby setting the stage for heart attacks and strokes [57, 60].

While LDL cholesterol takes the center stage in the narrative of CVD development, its detrimental impact is often heightened by accomplices such as high blood pressure, diabetes and smoking [57]. Furthermore, elevated levels of triglycerides, another type of blood fat, can compound the risk of CVDs, particularly when coupled with high LDL cholesterol [51]. This intricate web of interconnected factors underscores the multifaceted nature of CVD development, emphasizing the critical need for comprehensive approaches in addressing risk factors for effective prevention and management.

This global health challenge extends its reach to Africa, where CVDs contribute significantly to the continent's high mortality rate. Sub-Saharan Africa is home to 25 million people living with CVDs, and this prevalence is anticipated to rise by 25% by 2030 [21]. CVDs, claiming 13% of all deaths, stand as the leading cause of mortality in Africa, with a staggering 7.4 million people succumbing to premature deaths each year before the age of 70 [61].

Several factors contribute to this disproportionate burden in Africa. Inadequate access to healthcare services, poor preventative measures and late diagnosis and treatment options significantly contribute to the high mortality rate from CVDs [62–64]. Socioeconomic disparities, including poverty, unemployment and restricted access to good food and physical activity, contribute to the escalating prevalence of CVD risk factors in Africa [65–67]. Furthermore, the lack of public awareness about CVDs and their risk factors hampers early diagnosis and intervention, leading to complications and increased mortality [68, 69].

Cardiovascular diseases (CVDs) have risen as a pressing global health issue, and their impact on public health is profound. In 2008, CVDs accounted for nearly half of all reported deaths worldwide, highlighting their significant burden [64]. It is worth noting that this burden is particularly concentrated in low-to-middle-income countries [70], where healthcare resources and access to preventive measures are often limited.

A striking characteristic of CVDs is their association with an aging population, with more than 50 percent of CVD-related deaths occurring in individuals over the age of 70. This demographic shift places additional strain on healthcare systems, as older individuals often require specialized medical care and interventions. The African continent, with its vast and diverse population, is significantly impacted by the CVD epidemic. Sub-Saharan Africa alone reported approximately one million CVD-related deaths in 2013, comprising a substantial portion of global CVD fatalities and a noteworthy fraction of all deaths on the continent [71]. CVDs have become a major contributor to non-communicable disease-related mortality in Africa, reflecting a significant change in the region's disease landscape over the past few decades.

Moreover, there exists a notable disparity in CVD mortality between genders, with more than a 10 percent difference in death rates between females and males [71]. This variation underscores the need for gender-specific health strategies and interventions to address the unique risk factors and healthcare needs of women and men.

The evolving landscape of CVDs in Africa is shaped by epidemiological transitions and population dynamics, particularly in low-resource communities. Sub-Saharan Africa hosts a substantial proportion of the world's impoverished population, making it essential to develop strategies that are tailored to the region's specific socioeconomic and healthcare challenges [72]. Addressing the CVD burden in Africa necessitates a comprehensive and multi-faceted approach that takes into consideration the complex nature of factors contributing to this public health challenge.

### Lipid-related cardiovascular risk factors

In recent years, heightened attention has been directed towards cardiovascular risk factors linked to lipids, particularly low-density lipoprotein (LDL) and high-density lipoprotein (HDL) [26]. These factors, including elevated plasma triglycerides, have been extensively researched for prevention and treatment strategies.

LDL, a central player in cardiovascular health, undergoes a complex process resulting in the formation of small, dense LDL particles. This intricate process involves the exchange of lipids between triglyceride-rich lipoproteins and LDL, a phenomenon influenced by genetic traits. Aged small, dense LDL particles, with diminished protection against free radical attack, linger longer in the bloodstream. Their increased susceptibility to oxidative modification contributes to the formation of atherosclerotic plaques, as evidenced by studies like the Quebec Heart Study, highlighting a robust connection between small and dense LDL cholesterol concentration and the risk of coronary heart disease [73, 74].

Conversely, high-density lipoprotein (HDL) plays a protective role in cardiovascular health. Reduced levels of HDL are related to an elevated risk of cardiovascular diseases. HDL's atheroprotective function is achieved through the pathway of reverse cholesterol transport (RCT), wherein cholesterol is moved from peripheral tissues to the liver for elimination. This process involves crucial components such as lecithin-cholesteryl ester acyl-transferase (LCAT) and apolipoprotein Apo A1 [26, 75].

Beyond RCT, HDL exhibits various atheroprotective properties, including preventing the development of reactive oxygen species, inhibiting LDL oxidation, protecting endothelial cells from apoptosis and participating in inflammatory and apoptotic processes [76–81].

Maintaining healthy lipid levels is crucial for cardiovascular well-being. Factors such as estrogen, reduced body fat, moderate alcohol intake, strenuous exercise and certain medications like niacin and fibrates have been identified to positively impact HDL cholesterol levels and overall cardiovascular health [82, 83].

In unraveling the complexities of lipid dynamics, valuable insights emerge for developing comprehensive strategies aimed at addressing cardiovascular health and managing the associated risk factors linked to cardiovascular diseases.

### Atherosclerosis as a cardiovascular disease

Atherosclerosis is one of the most life-threatening cardiovascular diseases, which can affect individuals as early as their twenties. It contributes to an estimated 15.2 million deaths annually worldwide, making it a leading cause of mortality, as reported by the World Health Organization [24]. The initial stage of atherosclerosis is characterized by the accumulation of plaque in the arteries, particularly in large- and medium-sized vessels. This plaque primarily consists of cholesterol derived from low-density lipoproteins (LDLs), fibrin and calcium. The development of plaque can lead to ischemia due to the obstruction of blood flow and may result in the formation of thrombi when the plaque ruptures, leading to the blockage of blood vessels [25].

Atherosclerosis, or the formation of plaque, primarily occurs in the endothelium of arterial walls [26]. Under normal circumstances, the endothelium contributes to blood vessel dilation, reduces the growth of smooth muscle cells and prevents inflammatory responses [84]. However, in the context of atherosclerosis, dysfunction in the endothelium leads to reduced production of nitric oxide, a major vasodilator, resulting in increased vasoconstriction, heightened permeability and the uptake of LDL cholesterol by macrophages. This process leads to the formation of early lesions known as fatty streaks, the first visible sign of atherosclerosis [26].

Inadequate intake of antioxidants such as vitamin E, selenium and a diet low in fiber and unsaturated fats is dietary risk factor for atherosclerosis and dyslipidemias [85–89].

### Available remedies for the management of cardiovascular diseases

While statins continue to be the primary treatment for hypercholesterolemia, recent research has explored alternative remedies with promising results. These alternatives aim to address not only cholesterol levels but also potential side effects associated with statin therapy.

Dietary modifications, such as a lecithin-enriched diet, have shown promise in positively impacting lipoprotein metabolism and cholesterol homeostasis, potentially reducing overall cholesterol levels [17, 90]. Plant sterols and stanols, soluble fiber from sources like psyllium and oats, and adherence to the Mediterranean diet are additional dietary approaches with proven efficacy in lowering cholesterol and reducing cardiovascular risk [91–93].

Nutraceuticals and supplements, including red yeast rice, berberine, omega-3 fatty acids and Coenzyme Q10, offer additional options for managing hypercholesterolemia [94–97]. Lifestyle modifications, such as regular exercise, weight management and stress reduction techniques, also play a crucial role in lowering cholesterol levels and improving overall cardiovascular health [98–100].

While statins remain a valuable tool in managing hypercholesterolemia, alternative remedies offer promising options for individuals seeking additional support or experiencing side effects. A combination of dietary modifications, nutraceuticals, lifestyle changes and appropriate medical supervision can effectively manage cholesterol levels and reduce cardiovascular risk.

### Atherogenic lipoproteins

Several factors influence the atherogenic properties of cholesterol-containing lipoproteins in plasma. One critical factor is particle size, with smaller particles accumulating more rapidly in artery walls than larger particles. The size of the particle also determines its affinity for binding to the subendothelial matrix, with smaller particles binding more readily to proteoglycans [101]. Apolipoprotein B (Apo B), a protein found in lipoproteins, plays a significant role in this context, possessing multiple binding sites for proteoglycans and promoting the retention of lipoprotein particles within the subendothelial matrix, rendering lipoproteins containing Apo B atherogenic in nature [102].

Beyond particle size, additional factors contribute to the atherogenicity of cholesterol-containing lipoproteins. Remnant lipoproteins, resulting from the loss of their triglyceride cargo, exhibit prolonged circulation time and delayed clearance, increasing their interaction with the arterial wall and enhancing their atherogenic potential [103, 104].

Oxidative modification is another crucial aspect, with lipoproteins, particularly LDL, susceptible to modification by free radicals, leading to the formation of oxidized LDL (oxLDL). OxLDL is highly atherogenic, readily taken up by macrophages in the artery wall, triggering an inflammatory response and contributing to plaque formation [60, 105].

The composition and apoprotein profile further influence atherogenic potential. Lipoproteins enriched in triglycerides, such as VLDL remnants and chylomicron remnants, have higher atherogenic potential, while specific apolipoproteins associated with a lipoprotein particle, such as ApoC-III and ApoA-I, play distinct roles in influencing atherogenicity [106, 107].

Understanding these factors beyond particle size provides a comprehensive perspective on the complex interplay between lipoproteins and atherosclerosis. Targeting these factors through lifestyle modifications, medications and potentially novel therapeutic approaches can contribute to more effective strategies for preventing and managing cardiovascular diseases.

### Lecithin cholesterol acyltransferase (LCAT) activity in atherosclerosis and cardiovascular disease: insights from recent studies

For over 50 years, lecithin acyltransferase (LCAT) has been recognized as an enzyme capable of esterifying cholesterol in plasma. It plays a crucial role in HDL maturation and reverse cholesterol transport. Despite the enzyme's apparent benefits for cholesterol metabolism, its role in atherosclerosis pathogenesis remains debated [108].

Conflicting results arise from human studies on high-risk cardiovascular patients and the general population. Some studies suggest that LCAT activity could promote atherogenesis [109–112]. Conversely, other research indicates that LCAT deficiency and reduced HDL-C levels reduce the risk of atherosclerosis, coronary artery disease or ischemic heart disease [113–118]. Additionally, gender differences in LCAT activity have been reported, with women exhibiting higher LCAT activity having an increased risk of CAD compared to men [119, 120]. Table 1 shows a summary of studies showing the correlation between LCAT and atherosclerosis.

**Table 1:** Studies on LCAT and atherosclerosis (2000–2023)

Author(s), year	Study type	Population	Key findings
Yokohama et al., 2018	Longitudinal pilot study	Patient with one or more risk factor for ASCVD	Decreased LDL size, Increased formation of Triglyceride (TG)-rich lipoproteins (TRLs) Reduced risk of ASCVD may be associated with LCAT activity
Tani et al., 2016	Cross-sectional study	538 patients with at least risk factor for atherosclerosis	Increased formation of TRLs; Reduction in LDL particle size To reduce the risk of atherosclerotic cardiovascular disease, attention should be paid to LCAT activity, and not only quantitative change in LDL-C but also on LDL heterogeneity
Oldoni et al., 2018	Case–control	74 heterozygotes for LCAT mutations compared with 280 control	LCAT mutation in FED and FLD leading to increased or decreased risk for atherosclerosis is due to ability of LCAT to generate cholesterol esterification on APO-B
Calabresi et al., 2009	Case–control study	40 carriers of LCAT gene mutation and 80 matched controls	LCAT mutation carriers were found to have decreased cIMT with adjustment for other factors such as smoking, BMI, etc.
Dullart et al., 2010	Case–control study	116 men with CVD compared to 111 male controls	Subjects with CVD found to have greater LCAT activity, even at a given HDL-C level
Haase et al., 2012	Case–control study	Copenhagen City Heart Study (10,281), Copenhagen General Population Study	Genetically decreased HDL-C due to LCAT polymorphism does not correlate to increased risk of myocardial infarction
Ayyobi et al., 2004	Case–control study	FLD: 2 homozygotes and 8 heterozygotes for LCAT mutations	Ultrasound-guided cIMT greater in 6 out of 8 heterozygotes compared to controls, with plaques found in 4 heterozygotes
Hovingh et al., 2005	Case–control study	Five Dutch families with FED: 9 homozygotes and 47 heterozygous subjects with LCAT mutations, 58 family controls	Heterozygotes found to have increased ultrasound-guided cIMT, decreased HDL-C, increased triglycerides and C-reactive protein
Scarpioni et al., 2008	Case report	Case study on 1 FLD subject	Severe vascular disease reported in a FLD subject with triple-vessel CAD, angina at rest and PAD
Sethi, 2010	Case–control study	95 people with Ischemic heart diseases, 110 people without ischemic heart disease matched by age, gender and HDL-C	Individuals with IHD had lower LCAT activity in low and high HDL-C groups. IHD was also associated with high preB1-HDL concentration
Duivenvoorden et al., 2011	Case–control study	40 LCAT mutation carriers and age-matched controls	MRI and ultrasound-guided cIMT increased in LCAT mutation carriers
Van den Bogaard, 2012	Case–control study	45 LCAT mutation carriers and age-matched controls	LCAT mutation carriers had greater carotid-femoral pulse wave velocity that correlated positively with ultrasound and MRI-guided cIMT
Holleboom et al., 2010	Case–control study	933 men and women who acquired CAD compared with 1852 matched controls	LCAT level has no correlation with CAD risk. Highest quartile of LCAT concentration correlates with CAD in women
Calabresi et al., 2011		540 individuals with at least three cardiovascular risk factors	LCAT concentration has no correlation to whole cohort CAD risk. Increasing concentration of LCAT correlated to CAD in women
Gebhard et al., 2018	Case–control study	267 CAD patients and 96 control	Plasma LCAT mass concentration is upregulated in CAD patients and inversely related to plaque volume

In exploring the complex landscape of cholesterol metabolism, recent research has explored LCAT-independent pathways of cholesterol efflux, revealing potential mechanisms and clinical implications [108]. This adds a layer of complexity to our understanding of cholesterol homeostasis and suggests alternative routes for managing cholesterol levels beyond traditional pathways. LCAT deficiency has effects that go beyond the metabolism of HDL and can have wider clinical consequences due to its involvement in lipid metabolism. LCAT is essential for the esterification of cholesterol, which helps transport it in lipoproteins and contributes to the development of fully formed HDL particles. LCAT deficiency can cause a decrease in the ability to convert cholesterol into cholesterol esters, leading to the buildup of unbound cholesterol and triglycerides in lipoproteins that are circulating in the body. The disruption of lipid metabolism can make individuals more susceptible to certain cardiovascular problems, such as accelerated atherosclerosis, coronary artery disease and premature cardiovascular events. In addition, LCAT deficiency might present with symptoms such as corneal opacities, anemia and renal impairment, which demonstrate the widespread effects of poor lipid metabolism [121]. Hence, it is crucial to comprehend the wider ramifications of LCAT deficiency beyond HDL metabolism in order to effectively treat it clinically and devise specific therapeutic strategies.

LCAT deficiency has also garnered attention, extending beyond being perceived merely as a disorder of HDL metabolism. Contemporary investigations into LCAT deficiency have unveiled broader implications, shedding light on its intricate involvement in processes beyond HDL metabolism. This expanded perspective opens new avenues for exploring the role of LCAT and its deficiency in various aspects of lipid metabolism and related disorders.

One of such perspectives is the presence of lipoprotein X in LCAT deficiency conditions which might promote atherogenesis. Although its atherogenic potential is not fully confirmed, studies report extreme hypercholesterolemia mediated by lipoprotein X [122]. Also, some studies indicate that functional LCAT activity is not required for macrophage cholesterol efflux and RCT, as preβ-HDL acts as a cholesterol acceptor via ABCA1 or the SR-BI-dependent pathway [108, 109].

While there is growing interest in the role of LCAT and HDL in reducing atherosclerosis, leading to studies on various LCAT-related therapies, including recombinant LCAT and LCAT activation [123, 124], another perspective focuses on triglyceride-rich lipoproteins and low-density lipoprotein in atherosclerosis pathogenesis. Some studies suggest that increased LCAT activity may be linked to the formation of triglyceride-rich proteins and a concomitant reduction in LDL size [125, 126].

In a parallel line of inquiry, the emerging role of remnant lipoproteins in atherothrombosis has been a subject of interest [127]. The recognition of remnant lipoproteins as key players in this cardiovascular context adds depth to our understanding of atherothrombosis, potentially paving the way for targeted interventions that address these specific lipoprotein subtypes.

This evolving landscape of research highlights the dynamic nature of lipid-related studies, pushing the boundaries of our knowledge and encouraging a more comprehensive exploration of the intricate mechanisms that govern cholesterol metabolism and its implications for cardiovascular health.

### LCAT activity and the RCT system

**Fig. 2:**
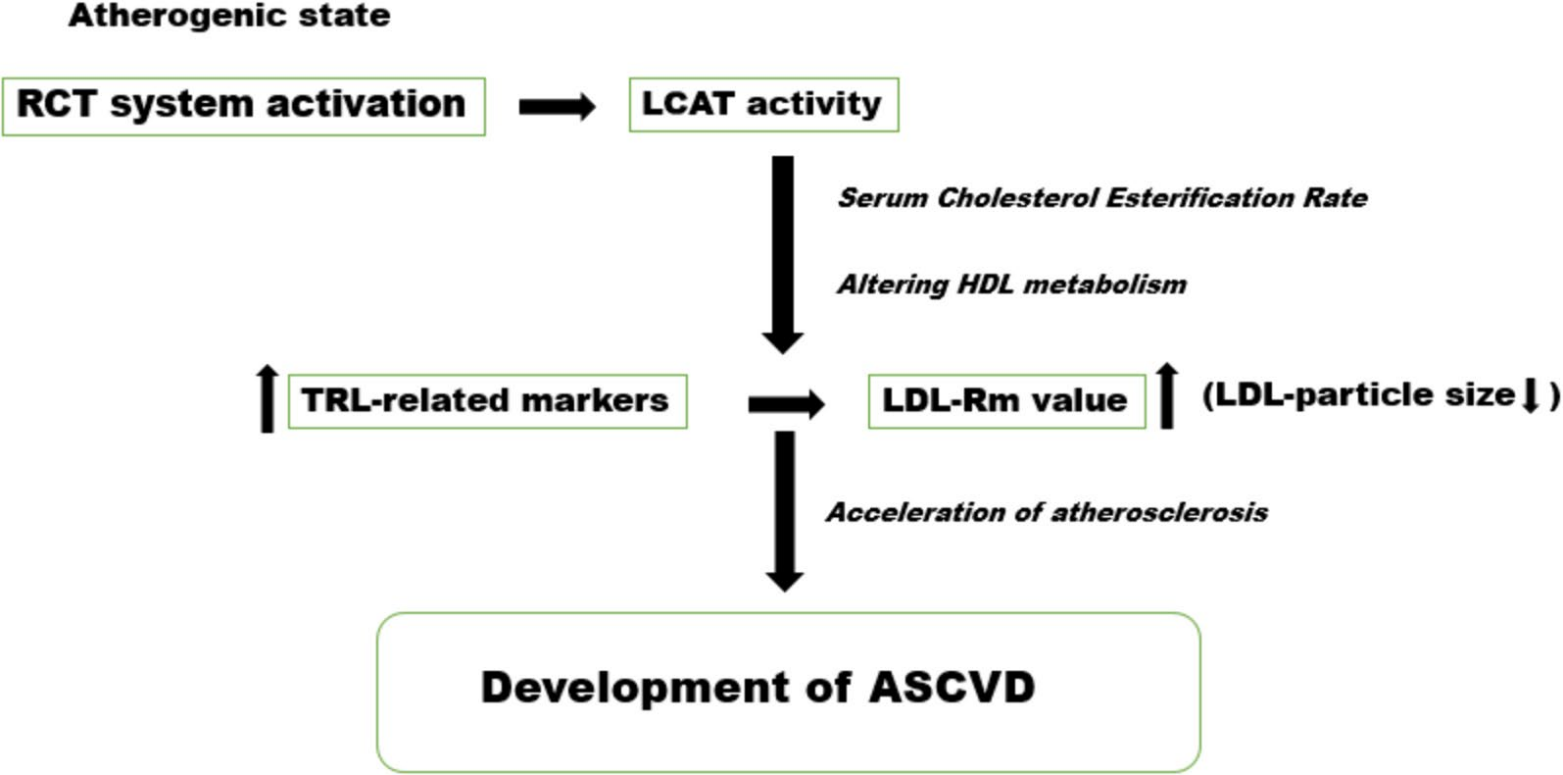
The association of LCAT activity as an indicator of LDL-particle size (LDL-Rm value) with ASCVD development [131]

Studies suggest that elevated LCAT activity may contribute to increased triglyceride-rich lipoproteins (TRLs) and, consequently, decreased LDL particle size. This is likely due to the actions of hepatic lipase and cholesteryl ester transfer protein (CETP), as supported by findings from some observational studies [125, 126]. These investigations reveal a positive association between LCAT activity and markers of TRL metabolism, indirectly influencing LDL particle size. However, it is essential to acknowledge that the reverse cholesterol transport (RCT) system is a complex network governed by more than just LCAT. Recent research implicates the interplay of additional enzymes, such as phospholipid transfer protein (PLTP) and CETP in shaping HDL's atherosclerogenic potential [128, 129]. Therefore, a holistic understanding of these intricate interactions is crucial for deciphering the multifaceted roles of LCAT and the RCT system in cardiovascular health. Figure 2 shows the association of LCAT activity and an indicator of LDL-particle size (LDL-Rm value) with ASCVD development. Figure 2 is a representation of the association of LCAT activity as an indicator of LDL-particle size (LDL-Rm value) with ASCVD formation.

### RCT and atherosclerosis

The efficiency of the reverse cholesterol transport (RCT) system in combating atherosclerosis is tightly linked to the state of lipid metabolism. Dyslipidemias, a significant contributor to atherosclerotic cardiovascular disease (ASCVD), can paradoxically trigger LCAT activation. This increased activity accelerates free cholesterol esterification, potentially altering the direction of atherosclerotic plaque suppression [130, 131].

Interestingly, studies have identified a negative association between LCAT mass concentration and intravascular ultrasonography-assessed plaque volume in patients with coronary artery disease (CAD) [131]. This suggests that cautiously modulating LCAT activity may promote a more balanced RCT function, potentially leading to plaque regression in CAD patients [131].

### The atherogenic cascade

This intricate mechanism comes into play in an atherogenic state, where RCT system activation, alongside increased LCAT activity measured using the rate of esterification of serum cholesterol via endogenous substrates, leads to alterations in HDL metabolism. These changes result in elevated serum levels of TRL-related markers and a reduction in LDL particle size, ultimately contributing to the development of ASCVD. These findings provide valuable insights into the complex interplay of factors in atherosclerosis and cardiovascular health, offering potential targets for therapeutic interventions in the ongoing battle against cardiovascular diseases.

## Conclusions

In the realm of cardiovascular health, lecithin emerges as a pivotal player in addressing the key risk factors—low-density lipoprotein (LDL), often regarded as the 'bad' cholesterol, and high-density lipoprotein (HDL), the 'good' cholesterol. Lecithin's role encompasses reducing LDL levels while simultaneously promoting the synthesis of HDL, thereby fortifying our defenses against cardiovascular ailments.

Notably, lecithin cholesterol acyltransferase (LCAT) takes the center stage in the intricate choreography of reverse cholesterol transport (RCT), an inherently anti-atherogenic process. At its core, RCT revolves around the removal of excess cholesterol by high-density lipoproteins (HDL) from cellular repositories.

### Recommendations

Building upon the insights gleaned from this review, several recommendations can be made to further our understanding and enhance cardiovascular health.

Given the potential benefits of lecithin in modulating cholesterol levels, there is a need for rigorous research aimed at determining the ideal lecithin dosage for individuals. Tailored dosages can provide a more targeted approach to managing cardiovascular risk factors. Also, the intricate association between increased LCAT activity and a reduction in LDL particle size in the context of atherosclerosis warrants further investigation. In-depth research is required to unravel the precise mechanisms underlying this relationship. A clearer understanding of these processes can allow for more targeted with effective interventions strategies.

Incorporating these recommendations into future research and medical practice can contribute to advancing our knowledge of cardiovascular health and developing more tailored approaches for prevention and treatment, ultimately benefiting individuals at risk of cardiovascular diseases.

## Data Availability

The datasets used and/or analyzed during the current study are available from the corresponding author on reasonable request.

## Abbreviations

LDL: Low-density lipoprotein
HDL: High-density lipoprotein
LCAT: Cholesterol acyltransferase
RCT: Reverse cholesterol transport
TRL: Triglyceride-rich lipoprotein
ASCVD: Atherosclerotic cardiovascular disease
CVD: Cardiovascular diseases
Apo B: Apolipoprotein B
Oxldl: Oxidized LDL
CETP: Cholesteryl ester transfer protein
PLTP: Phospholipid transfer protein
